# Socioeconomic and Demographic Determinants of COVID-19 Vaccination Attitudes and Uptake Among Residents of Western Attica, Greece: A Cohort Study

**DOI:** 10.7759/cureus.75983

**Published:** 2024-12-18

**Authors:** Georgios F Fragkiadakis, Maria Koutoula

**Affiliations:** 1 Social Sciences School, Hellenic Open University, Chania, GRC; 2 Social Sciences School, Hellenic Open University, Athens, GRC

**Keywords:** covid-19, greece, health equity, public health, socioeconomic factors, vaccine hesitancy

## Abstract

The COVID-19 pandemic posed a major public health challenge during its early stages, and vaccine distribution played a critical role in the initial response. This cohort study examines the socioeconomic and demographic factors influencing attitudes towards COVID-19 vaccination in Western Attica, Greece. The data was collected in two phases: In the first phase (December 2021-January 2022), 269 people who had initially refused the vaccination were surveyed. By June 2022, 207 people had agreed to be vaccinated, while 62 people remained unvaccinated, with 48 people giving reasons for their refusal. Statistical analysis showed that higher income and higher education levels significantly increased acceptance of vaccination (p=0.003 for income; p=0.001 for education), while participants with chronic conditions were also more likely to be vaccinated (p=0.024). External factors such as government-imposed fines motivated 29.47% of participants, and 26.57% were influenced by personal experiences with COVID-19. These findings emphasise the need for targeted public health interventions to reduce vaccination hesitancy among lower socioeconomic groups and focus on vulnerable populations, particularly those with chronic health conditions. The study adds to the growing body of research on vaccine hesitancy and provides evidence for policy strategies to improve immunisation coverage in underserved populations.

## Introduction

The COVID-19 pandemic, which emerged at the end of 2019, quickly became one of the biggest public health challenges worldwide, prompting the World Health Organization (WHO) to declare it a pandemic in March 2020. Measures such as lockdowns and travel restrictions were quickly put in place to contain the virus, which spread rapidly and overwhelmed healthcare systems worldwide, especially in Europe [1-3].

In Greece, the virus was initially contained through early intervention, but the healthcare system, already under severe strain due to ongoing austerity measures, came under pressure. In addition to austerity, the healthcare system was further strained by an aging population, a high prevalence of chronic diseases, and limited healthcare resources, which collectively weakened its capacity to respond to health crises effectively. This situation underscored the long-standing weaknesses of the healthcare system and highlighted the need for coordinated support at the national and EU levels, such as the EU4Health program, which strengthened healthcare systems and vaccine preparedness [4-8].

In the past, vaccines have played an important role in combating infectious diseases and improving public health by reducing mortality from diseases such as smallpox and measles. Today, vaccines continue to be crucial in controlling infectious diseases worldwide, protecting populations and preventing outbreaks. The COVID-19 pandemic has reinforced this role and made the rapid development and global distribution of vaccines an urgent international priority [9-12].

Reluctance to vaccinate, especially among socioeconomically disadvantaged groups, is a significant barrier to achieving herd immunity [13]. This problem is closely linked to socioeconomic inequalities, as marginalized populations often have limited access to health care and concerns about vaccine safety and efficacy, which are exacerbated by misinformation during the pandemic [13-15]. Vaccination hesitancy is influenced by complex factors, including misinformation, mistrust of health authorities, and deeply held cultural and religious beliefs [16,17].

Globally, vaccine hesitancy is influenced by regional cultural and socio-political factors. In Eastern Europe, anti-vaccination movements have weakened public confidence in the safety of vaccines, exacerbated by economic inequalities and limited access to healthcare in rural areas [18-20]. In Asia, e.g., Indonesia, religious beliefs and misinformation have significantly impacted vaccine acceptance, particularly in underserved regions with low health literacy [19,20]. The spread of misinformation, especially on social media, has further fueled vaccine skepticism worldwide. For example, in South Korea, misinformation led to lower vaccination rates due to fears of possible side effects, while in Sri Lanka, a study found that 37.8% of respondents delayed or refused vaccination mainly due to misunderstanding and fear of adverse effects [21,22].

Western Attica, Greece, illustrates how such global trends intersect with local challenges. This region, characterised by persistently low vaccination rates, faces significant socio-economic disparities, including high unemployment, low incomes and limited access to health services. These issues, combined with widespread misinformation, contribute to vaccine hesitancy and low immunisation coverage [23,24]. Addressing these challenges is critical to developing targeted public health interventions that promote health equity and ensure equitable access to vaccines and accurate information for all socioeconomic groups.

The primary objective of this study is to investigate the socioeconomic and demographic factors that influence vaccination decisions among residents of Western Attica, Greece. Specifically, the study aims to assess knowledge and attitudes toward vaccination, focusing on perceptions of vaccine safety and effectiveness. By analyzing these factors, the research seeks to identify barriers to vaccination and provide actionable insights for targeted public health interventions to address health disparities in underserved populations. To achieve this, the study is structured in two phases. The first phase examines participants' initial reluctance to receive the COVID-19 vaccine, while the second phase analyzes changes in attitudes among those who eventually accepted vaccination. This two-phase design allows for a comprehensive understanding of the evolving factors that shape vaccination behavior over time.

## Materials and methods

Study design

In this study, a Strengthening the Reporting of Observational Studies in Epidemiology (STROBE) cohort reporting design [25] with two phases of data collection was used to investigate the socioeconomic and demographic factors influencing COVID-19 vaccination decisions and to track changes in the attitudes of residents of Western Attica, Greece, over time.

In the first phase, two structured online questionnaires were administered to participants who had not yet been vaccinated against COVID-19 at that time. The first questionnaire dealt with general attitudes towards vaccination, while the second captured specific attitudes towards COVID-19 vaccination. Participants’ responses were analyzed based on their demographic and socioeconomic characteristics.

In the second phase, conducted six months later, the same participants were asked to complete the second questionnaire again, focusing on attitudes towards COVID-19 vaccination. In this phase, both those who had subsequently decided to be vaccinated and those who had remained unvaccinated were interviewed, allowing an analysis of changes in attitudes over time.

The data was collected in both phases using structured online questionnaires. Participants received detailed information about the study and its objectives via the online questionnaire platform. Informed consent was obtained digitally, as participants had to read the consent form and confirm their agreement before proceeding to complete the questionnaire.

Selection and description of participants

The first phase of the survey was conducted from 01 December 2021 to 31 January 2022 with a sample of 269 adults who had not yet received the COVID-19 vaccine. The sample size of 269 adults was selected based on the study's time limitations and the practical constraints of participant recruitment. While the sample size was not calculated using a formal formula, it was determined based on the practical constraints of a two-phase study conducted during the pandemic. We acknowledge this as a limitation and attribute it to the challenges of engaging participants in a period marked by psychological strain and reluctance to participate. Participants were recruited through targeted invitations shared via social media platforms, specifically addressing unvaccinated individuals residing in Western Attica. This approach, while relying on convenience sampling due to the self-selection process, ensured that the study's inclusion criteria were met. Eligibility criteria included also being at least 18 years old and having given informed consent. The second phase of data collection (from 01 June 2022 to 30 June 2022) included 207 of these individuals who eventually opted to be vaccinated. Of the 62 remaining unvaccinated participants, 14 declined further participation, while 48 continued the study and provided information on their reasons for not being vaccinated. The inclusion of demographic variables such as age, gender, education and income ensured a diverse sample of unvaccinated individuals from Western Attica. Although no formal stratification or quotas were applied, the demographic data presented in the following section show a broad representation of key socio-demographic characteristics, allowing for meaningful analysis of vaccination attitudes and decisions. 

Data collection and measurements

The data were collected using a structured online questionnaire distributed via email and social media platforms to ensure accessibility for the target population in Western Attica. The questionnaire was designed to assess attitudes towards vaccination, vaccination decisions, and the influence of demographic factors. To align with the study's objectives and the cultural characteristics of the target population, the questionnaires were adapted linguistically and contextually from validated instruments (Sarathchandra et al. [26], Konstantinou et al. [27]). These adaptations included translation into Greek and ensuring cultural relevance. However, the process did not involve a full transcultural adaptation or an independent psychometric validation. It is important to note that the adapted scale, originally developed by Sarathchandra et al. [26] and later utilized by Konstantinou et al. [27], demonstrated high internal consistency reliability in previous studies. Nevertheless, a full validation process of the adapted scale was not conducted in the study by Konstantinou et al. [27].

To facilitate completion of the questionnaire and improve participant understanding, the original 7-point Likert scale from the referenced studies was adapted to a 5-point scale (1 = strongly disagree, 5 = strongly agree). This adjustment aimed to reduce cognitive burden and enhance response clarity, particularly in a population with varying levels of familiarity with survey instruments.

Demographic Information

In this section, age, gender, income, education, employment status and specific reasons for opposing vaccination were recorded. The reasons for refusing vaccination were included in the demographic section to provide an initial overview of the sample. Detailed analyses of vaccination attitudes are presented in the following sections.

General Attitudes Towards Vaccination

Following Sarathchandra et al. (2018) [26], a modified questionnaire was developed to assess attitudes towards vaccination using a five-point Likert scale (1 = strongly disagree, 5 = strongly agree). The questionnaire consisted of 20 questions to assess beliefs about the safety, necessity and social benefits of vaccination.

COVID-19 Vaccine-Specific Attitudes

Following the study by Konstantinou et al. (2021) [27], this section included five specific questions on beliefs and attitudes towards the COVID-19 vaccine. To make it easier for the target group to answer, the original seven-point Likert scale was converted to a five-point Likert scale (1 = strongly disagree, 5 = strongly agree). These questions focussed on perceived safety, effectiveness and willingness to be vaccinated with COVID-19 in the future.

In the second phase, participants who had chosen to be vaccinated were asked to describe their reasons for choosing the vaccine. Non-vaccinated participants had the opportunity to explain their reasons for continuing to refuse. Open-ended responses were analysed thematically to identify the most important motivations and concerns. The thematic analysis was conducted independently by two researchers, ensuring consistency and reliability. Missing responses for specific survey items were minimal (<5%) and were excluded pairwise during statistical analyses to ensure the integrity of the results.

Statistical analysis

Descriptive statistics, including mean values (MV) and standard deviations (SD), were calculated for the demographic data and Likert scale responses. T-tests, including paired t-tests, were used to assess differences between responses in Phase 1 and Phase 2 and to evaluate the statistical significance of observed changes in vaccine attitudes across different socioeconomic groups. The results confirmed significant differences across all groups, with T-statistics and p-values presented in the Appendix 1.

One-way ANOVA tests were conducted to compare vaccine attitudes across groups defined by categorical variables such as educational level, marital status, employment status, sector of employment, and monthly income. The assumptions of ANOVA were evaluated through visual inspection of data distributions, and the method was deemed appropriate for this analysis.

Logistic regression models were used to determine the influence of variables such as income, education, and chronic health conditions on vaccine acceptance. Odds ratios (OR) and 95% confidence intervals (CI) were reported for significant predictors.

A sensitivity analysis was performed by categorising income into two broader groups: €0-1000 and €1000-2000. Responses from the extreme income categories (€0-500 and €2000+) were excluded due to their low representation (n=12 and n=10 respectively) to ensure more reliable comparisons between groups with sufficient sample size.

Statistical significance was defined as p < 0.05 for all tests.

Ethical approval

This study was approved by the Faculty of Social Sciences of the Hellenic Open University, with Ethics Committee approval number 170612, dated 21 September 2021.

## Results

Results of the first phase: attitudes and hesitation among unvaccinated people

The first phase of the study focused on individuals who had not yet been vaccinated with the COVID-19 vaccine and aimed to understand the socioeconomic and demographic factors influencing vaccination hesitancy.

The sample comprised 269 participants, as shown in Table 1, with the majority being female (62.1%) and university graduates (65%). Most participants were employed (82.1%), both in the public and private sectors. A significant proportion reported having a monthly income of between €500 and €1000 (48.3%), while 36.1% earned between €1000 and €1500. In terms of health status, 27.2% of participants were receiving systemic drug therapy. Although the study aimed to capture a diverse sample, participants with higher levels of education are overrepresented and the proportion of participants undergoing systematic therapy is lower. These imbalances reflect the nature of the sample used and may limit the generalisability of the results for these specific subgroups. Nevertheless, the trends observed provide valuable insights into how education and chronic health conditions influence attitudes and decisions to vaccinate.

**Table 1:** Demographic data of citizens who participated in the survey (first phase) N represents the number of values counted and Frequency represents the percentage value

Demographic Categories		N	Frequency %
Gender	Male	100	37.2%
	Female	167	62.1%
	Not answered	2	0.7%
Age groups	18-34	57	21.2%
	35-49	101	37.5%
	50-64	93	34.6%
	65+	18	6.7%
Educational level	Primary education	14	5.2%
	Secondary education	80	29.7%
	Higher education	133	49.4%
	Master's degree/PhD	42	15.6%
Employment status	Employee	220	82.1%
	Unemployed	15	5.6%
	Student (higher ed./Univ.)	12	4.5%
	Retired	18	6.7%
	Student	3	1.1%
Employment sector	Public sector	100	41.5%
	Private sector	104	43.2%
	Self-employed	37	15.4%
Nationality	Greek	269	100.0%
Monthly income	0	12	4.6%
	500-1000	127	48.3%
	1000-1500	95	36.1%
	1500-2000	19	7.2%
	>2000	10	3.8%
Systemic drug therapy	Yes	73	27.2%
	No	196	72.8%

The descriptive analysis of participants' attitudes towards vaccines (see Table 2) shows that the safety and efficacy of vaccines are generally viewed favourably. Statements such as "Vaccines are effective in preventing diseases" and "Vaccines are a major advancement for humanity" had high mean scores (mean = 4.1, SD = 0.7 and mean = 4.2, SD = 0.7, respectively). However, safety concerns persisted, as reflected in lower mean scores for beliefs such as "Vaccines contain mercury in dangerous amounts" (mean = 2.5, SD = 0.8) and "Vaccines cause autism" (mean = 2.3, SD = 0.8). Attitudes towards government regulations were mixed, with moderate support for mandated vaccinations (mean = 2.9, SD = 1.2), but stronger support for following government guidelines to protect public health (mean = 3.5, SD = 1.0).

**Table 2:** Descriptive results for citizens' attitudes and perceptions toward vaccines (1=strongly disagree, 5=strongly agree) Mean: Average response score. SD: Standard Deviation. Responses were measured on a five-point Likert scale, where 1 indicates 'strongly disagree' and 5 indicates 'strongly agree'.

Survey Statements	Mean ± SD
Vaccines are safe.	3.9 ± 0.8
Vaccines contain mercury in dangerous amounts.	2.5 ± 0.8
Vaccines contain dangerous ingredients	2.4 ± 0.8
Vaccines cause autism.	2.3 ± 0.8
Some vaccines are unnecessary since they target relatively harmless diseases.	2.7 ± 1.0
Diseases provide better immunity than vaccines do.	2.9 ± 1.1
Vaccines are effective at preventing diseases.	4.1 ± 0.7
Many of the illnesses that vaccines prevent are severe.	4.2 ± 0.6
We give children the right number of vaccines.	3.7 ± 0.8
The timing of the current vaccination schedule is appropriate.	3.5 ± 0.8
We give vaccines to children when they are too young.	3.9 ± 0.8
We give children too many vaccines.	2.5 ± 0.8
I am morally opposed to vaccinating my child.	2.1 ± 1.0
Vaccines conflict with my belief that children should use natural products and avoid toxins.	2.3 ± 1.0
Vaccines are a major advancement for humanity.	4.2 ± 0.7
Vaccines are disgusting to me.	1.7 ± 0.8
The government should not force children to get vaccinated to attend school.	2.9 ± 1.2
My right to consent to medical treatment means that vaccinations should always be voluntary.	3.1 ± 1.1
To protect public health, we should follow government guidelines about vaccines.	3.5 ± 1.0
It is legitimate for government to mandate vaccinations.	2.9 ± 1.2

As shown in Table 3, participants' attitudes towards the COVID-19 vaccine varied. Moderate optimism was observed regarding the ability of the vaccine to stop the pandemic (mean = 3.3, SD = 1.1), while there was strong support for the global availability of the vaccine (mean = 4.1, SD = 0.9). Opinions on mandatory vaccination were mixed, with a mean score of 2.9 (SD = 1.2). Participants also showed a moderate willingness to be vaccinated annually with the COVID-19 vaccine (mean = 3.1, SD = 1.1), and experience with the pandemic led to only limited changes in vaccination attitudes (mean = 2.7, SD = 1.0).

**Table 3:** Descriptive results for citizen attitudes toward Covid-19 vaccine (1=strongly disagree, 5=strongly agree) Mean: Average response score. SD: Standard Deviation. Responses were measured on a five-point Likert scale, where 1 indicates 'strongly disagree' and 5 indicates 'strongly agree'.

Survey Statements on COVID-19 Vaccination	Mean ± SD
I believe that the discovery of a vaccine against SARS-CoV-2 will stop the pandemic of the disease COVID -19	3.3 ± 1.1
I believe that the vaccine against SARS-CoV-2 should be made available in all countries of the world	4.1 ± 0.9
I believe that the SARS-CoV-2 vaccine should be mandatory for all people who can be vaccinated.	2.9 ± 1.2
If a seasonal vaccine against the SARS-CoV-2 virus that causes the disease COVID -19 is available, I will take it every year.	3.1 ± 1.1
I changed my mind about vaccination during this experience with the pandemic COVID -19.	2.7 ± 1.0

As described in the supplementary material (Appendix 2), there were no significant differences in the perception of vaccine safety between education levels (p = 0.311). However, participants with higher levels of education rated the effectiveness and necessity of vaccines more favourably (p = 0.006). In addition, education influenced views on the value of vaccination, with participants with higher levels of education expressing a more favourable opinion (p = 0.003).

As shown in Table 4, participants undergoing systemic drug therapy were generally more positive about the safety, efficacy and necessity of vaccines than those who did not receive therapy. Statistically significant differences were found in these areas (p < 0.05). In addition, participants on therapy showed greater acceptance of vaccine selection and scheduling, as well as more favourable attitudes towards government vaccination requirements and the COVID-19 vaccine (p < 0.05 for all comparisons). These results suggest that people receiving systemic drug therapy perceive vaccines more favourably across multiple dimensions.

**Table 4:** Results of the t-test comparing citizens' attitudes and perceptions regarding vaccines and systemic drug therapy Mean: Average response score. S.D.: Standard Deviation, t is the t-test value and P: is the Probability Value where a p value < 0.05 was considered statistically significant

Vaccine Attitudes and Perceptions	With Systemic Therapy	Without Systemic Therapy	t	p
	Mean ± SD	Mean ± SD		
Perceived safety	3.9 ± 0.6	3.7 ± 0.7	2.175	0.030
Perceived effectiveness and necessity of vaccines	3.8 ± 0.5	3.6 ± 0.7	2.181	0.030
Acceptability of vaccine selection and scheduling	3.8 ± 0.4	3.6 ± 0.7	2.115	0.035
Positive values	4.2 ± 0.6	3.9 ± 0.7	2.267	0.024
Perceived legitimacy of authorities to mandate vaccinations	3.4 ± 0.8	3.0 ± 0.9	2.639	0.009
Attitude toward Covid-19 vaccine	3.7 ± 0.8	3.3 ± 0.9	2.950	0.003

Results from the second phase: attitudes and behaviors among vaccinated individuals

In the second phase, 207 participants who had initially hesitated ultimately received the COVID-19 vaccine (Appendix 3). Most of these participants were female (n = 120, 57.97%), with a significant proportion over the age of 60 (n = 82, 39.61%). Additionally, many had a university degree (n = 102, 49.28%), some Master's or PhD degree (n = 32, 15.46%) and a large percentage reported a monthly income between €500 and €1500 (n = 168, 81.16%). Meanwhile, 18.85% (n = 48) remained unvaccinated, and 5.21% (n = 14) opted out of further participation in the study.

Appendix 4 provides a breakdown of the reasons for vaccine refusal in both phases. In the first phase, the primary reasons cited were concerns about vaccine safety (67%) and personal beliefs (12%). By the second phase, although the number of unvaccinated participants had declined, these reasons persisted, with safety concerns remaining the most frequently mentioned. While no significance testing was conducted due to the relatively small sample size in Phase 2 (n = 48), these results provide valuable insights into changes in vaccine hesitancy factors over time. 

**Figure 1:**
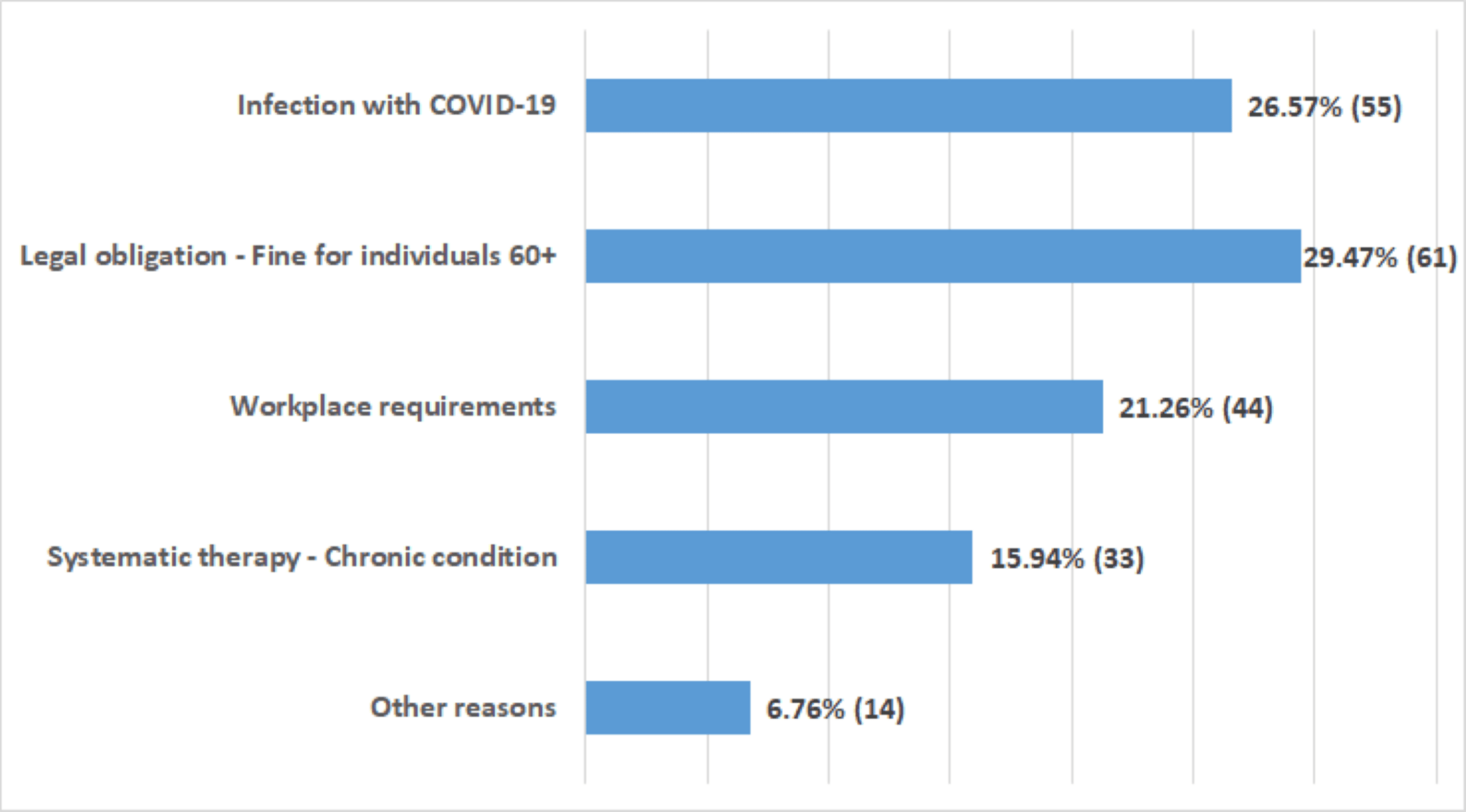
Drivers of COVID-19 vaccination acceptance The values are expressed as a percentage (%) and show the proportion of positive reactions in relation to the total number of people vaccinated. The values in brackets are expressed as number of responses (n).

Figure 1 illustrates the main reasons why participants chose to receive the COVID-19 vaccine. The most common reason was the legal requirement for individuals over 60 years old (n = 61, 29.47%), followed by previous infection with COVID-19 (n = 55, 26.57%) and workplace requirements (n = 44, 21.26%). Chronic illness was reported by 15.94% (n = 33) of participants, while 6.76% (n = 14) cited other reasons for vaccination.

The logistic regression analysis shown in Table 5 identifies several key factors that influence the likelihood of COVID-19 vaccination. Pearson correlation analyses (Appendix 5) show significant associations between various vaccination-related attitudes, including perceived safety, efficacy and legitimacy of vaccination requirements. These results suggest that attitudes towards vaccines are interrelated, which was taken into account in the selection of variables, including demographic factors, for the regression model. Additional analyses, available upon request, examine explicit relationships between demographic variables and vaccine attitudes.

**Table 5:** Logistic regression for predicting vaccination Odds Ratio (OR): Represents the likelihood of vaccination (OR > 1 indicates increased likelihood), 95% CI: Confidence interval for the OR; statistical significance if it does not include 1, p-value: Indicates statistical significance; p < 0.05 is considered significant. Beta values (β): The natural logarithm of the OR, representing the log-odds of vaccination.

Variable	Odds Ratio (OR)	Beta Value (β)	95% CI	p-value
Income (0 - 1,500 €)	1.45	0.37	1.12 - 1.89	0.003
Education (Primary/Secondary)	0.75	-0.29	0.56 - 1.02	0.001
Employment Status	1.22	0.20	0.90 - 1.65	0.112
Chronic Illness	1.88	0.63	1.43 - 2.47	0.001
Age	1.32	0.28	1.05 - 1.67	0.021

Table 5 shows that higher income (above €1500) significantly increases the likelihood of vaccination (OR = 1.45, 95% CI: 1.12-1.89, p = 0.003; β = 0.37), suggesting that financial stability improves access to vaccines and confidence in their efficacy. Education also proved to be a decisive factor: people with primary or secondary education were less likely to be vaccinated than those with higher education (OR = 0.75, 95% CI: 0.56-1.02, p = 0.001; β = -0.29). Chronic diseases proved to be a strong predictor of willingness to be vaccinated (OR = 1.88, 95% CI: 1.43-2.47, p = 0.001; β = 0.63), which is probably due to an increased perception of risk. Age also had a positive influence on the likelihood of vaccination, with older people being more likely to be vaccinated (OR = 1.32, 95% CI: 1.05-1.67, p = 0.021; β = 0.28). The referent age group for this variable was '18-34 years,' which represents the youngest cohort in the sample. This choice was made to provide a meaningful baseline for comparisons with older age groups, given the notable differences in vaccination attitudes and behaviors observed between younger and older populations in existing literature. Employment status did not show a significant effect on vaccination likelihood (OR = 1.22, 95% CI: 0.90-1.65, p = 0.112; β = 0.20). The referent category for employment status was 'unemployed,' representing individuals without active employment. This choice allows for meaningful comparisons against employed and retired individuals to assess the potential impact of employment on vaccination likelihood. The unemployed group, although smaller in size (5.6%), provides a distinct baseline for examining differences between actively working and non-working populations.

The sensitivity analysis confirmed the robustness of the relationship between income level and willingness to vaccinate, even when income was categorised more broadly. For example, willingness to vaccinate remained high at 87% for participants in the €1000-2000 range, compared to 71% for participants in the €0-1000 range. These results emphasise the impact of socioeconomic inequalities on health outcomes, with higher earners more likely to accept the COVID-19 vaccine. 

Our findings demonstrate changes in attitudes toward the COVID-19 vaccine across different socio-economic groups, as shown in Table 6. Participants with higher income (over €1,500) reported more positive views on the vaccine in both phases (Phase 1: 4.3; Phase 2: 4.8) compared to those with lower income (Phase 1: 2.8; Phase 2: 3.5). Similarly, individuals with tertiary education exhibited higher confidence in the vaccine (Phase 1: 4.5; Phase 2: 4.9) compared to those with primary or secondary education (Phase 1: 3.2; Phase 2: 4.0). Employed participants also reported more favourable attitudes (Phase 1: 4.2; Phase 2: 4.7) than retirees or unemployed individuals (Phase 1: 3.7; Phase 2: 4.3).

**Table 6:** Change in attitudes toward vaccination by socio-economic factors Responses were measured on a five-point Likert scale, where 1 indicates "strongly disagree" and 5 indicates "strongly agree." The data represent the average scores for each category. Phase 1: Responses prior to vaccination, Phase 2: Responses following vaccination

Statement	Phase	Income below 1,500 €	Income over 1,501 €	Primary & Secondary Education	Tertiary Education	Retirees / Unemployed	Employed
Positive views on COVID-19 vaccine	1	2.8	4.3	3.2	4.5	3.7	4.2
	2	3.5	4.8	4.0	4.9	4.3	4.7
Belief that the discovery of the vaccine will stop the pandemic	1	2.6	4.1	2.8	4.5	3.2	3.9
	2	3.3	4.7	3.6	4.8	3.9	4.5
Changed opinion about vaccination during the pandemic	1	2.4	3.8	2.2	4.0	3.1	3.5
	2	3.1	4.2	3.4	4.4	3.8	4.1
If a seasonal vaccine for SARS-CoV-2 is available, I will take it	1	2.2	3.7	2.3	3.8	2.5	3.0
	2	2.9	4.1	3.0	4.2	3.4	4.0

To validate these observed differences, paired t-tests were conducted, and the results, presented in Appendix 1, confirm that these changes are statistically significant across all groups. Specifically, participants with lower income (below €1,500) showed a significant increase in positive views (T = -5.46, p = 0.004), as did those with higher income (T = -9.92, p = 0.022). For individuals with tertiary education, the improvement was highly significant (T = -15, p = 0.006), while participants with primary or secondary education also showed meaningful progress (T = -7.89, p = 0.042). Similarly, both employed (T = -7.89, p = 0.004) and retired or unemployed individuals (T = -11.52, p = 0.001) demonstrated statistically significant improvements in their attitudes. These findings corroborate the upward trend in vaccine acceptance observed in Table 6 and emphasize the consistent impact across socio-economic groups.

## Discussion

This study provides valuable insights into the socioeconomic and demographic factors influencing attitudes towards COVID-19 vaccination and its uptake in Western Attica, Greece. The results reveal significant differences in vaccination behaviour that are closely linked to health inequalities. These findings emphasise the need for targeted public health interventions to remove barriers for vulnerable populations and promote equitable access to vaccines.

Our analysis shows that people with higher income and education levels are more likely to accept COVID-19 vaccination and feel safe. Specifically, participants with a higher level of education (including tertiary, Masters, and PhD degrees) demonstrated significantly more positive attitudes towards vaccination, with a statistically significant odds ratio of 0.75 for primary/secondary education versus higher education (p = 0.001). These findings highlight the role of socio-economic advantages in improving trust in health systems and access to credible vaccine-related information. Previous research has highlighted the pivotal role of education and income in shaping attitudes toward vaccines, indicating that individuals with higher socio-economic status exhibit greater trust in vaccines due to better access to accurate information and health services [28]. Socio-economic determinants have been identified as crucial predictors of vaccine acceptance [29], while geopolitical and socio-economic factors have also been shown to influence vaccine dissemination and acceptance [30]. Notably, our study echoes these conclusions, as participants in Western Attica with higher income and education expressed greater confidence in the safety and efficacy of COVID-19 vaccines.

In addition, socioeconomic inequalities that affect vaccine hesitancy are well documented [31,32]. Distrust in healthcare systems, concerns about vaccine safety, and misinformation are common reasons for vaccine hesitancy among low-income and marginalized populations. Similar trends were evident in our study, in which low-income participants showed higher levels of hesitancy, highlighting the need for targeted public health interventions tailored to the socioeconomic context of Western Attica.

Although our study focuses on Western Attica, similar challenges have been reported in economically disadvantaged groups across Europe. For example, research from Central and Eastern Europe highlights that distrust of healthcare systems and government interventions, as well as safety concerns, are significant barriers to vaccination in these regions [33-35]. While these findings are not directly comparable due to differences in sociopolitical and healthcare systems, they underscore the universal nature of socioeconomic barriers to vaccination. Addressing these challenges requires locally tailored, community-based campaigns to build trust and combat misinformation.

Our study highlights the influence of chronic health conditions on vaccination decisions. Among the study population, 27.2% of participants were undergoing systemic therapy. These participants reported higher confidence in the safety of vaccines, with a mean safety perception of 3.9 (SD = 0.6) compared to 3.7 (SD = 0.7) for those not receiving therapy (p = 0.024). Global research highlights that people with chronic diseases, such as diabetes or cardiovascular problems, face significantly higher risks of severe COVID-19 complications. This increased vulnerability underpins targeted vaccination strategies and may contribute to higher vaccine acceptance among these groups [36,37].

Research on vaccine strategies in low- and middle-income countries emphasises that addressing specific health concerns in people with chronic conditions can significantly improve vaccine uptake [38,39]. This is consistent with our findings and emphasises the importance of health campaigns targeting vulnerable groups, especially people with chronic diseases. Tailored communication about the increased risks of COVID-19 for these populations has been shown to increase confidence in the vaccine and vaccination uptake [40]. Contextualised vaccine communication that addresses the specific needs of people with chronic conditions is critical to reducing inequalities and promoting equitable health outcomes.

Our study also showed the impact of mandatory vaccination and government fines on increasing vaccination rates, especially among the elderly population. Nearly 29.47% of participants indicated that government fines were the main motivation for vaccination, while 26.57% cited personal experience with COVID-19 as an important factor. These findings are consistent with previous research that has shown that government regulations can encourage compliance, even when there are ethical concerns. However, studies suggest that while coercive regulations are effective in the short term, they can undermine public trust and lead to increased resistance to vaccination and other health interventions [41,42]. Behavioural psychology research also points to the risk of a backlash, particularly among people who already distrust the authorities. To mitigate these challenges, health authorities need to find a balance between regulatory measures and confidence-building measures. Transparent and evidence-based communication is essential to ensure that regulations are perceived as measures to protect public health and not as punitive measures.

Finally, the changed attitudes of initially hesitant individuals after vaccination emphasise the transformative role of personal experience in reducing hesitancy. Research consistently shows that vaccine hesitancy is not a fixed trait, but develops based on personal and social experiences. For example, a longitudinal US study found that almost a third of people who were initially hesitant eventually decided in favour of vaccination, underlining the dynamic nature of vaccination decisions [43]. This change is often due to observations of vaccine safety and efficacy, as well as the influence of trusted community members who act as advocates for vaccination. Our findings are consistent with this trend and show that the confidence of vaccinated participants, particularly those from lower socioeconomic backgrounds, has increased. Furthermore, there is evidence that providing accessible, clear and culturally relevant information is crucial to influence attitudes before and after vaccination [44]. In addition, emphasising the collective benefits of vaccination and removing specific barriers - such as logistical and informational challenges - can encourage higher uptake. These findings emphasise the need for continued community engagement and proactive efforts by healthcare providers to build trust and disseminate reliable information. These efforts should be designed to resonate with diverse populations, particularly in vulnerable or underserved communities. This will ensure that immunisation campaigns are both inclusive and effective in bridging gaps in confidence and access.

The analysis of the 48 participants in the second phase who did not accept the COVID-19 vaccine provides valuable insights into the reasons for vaccination reluctance. Of these individuals, 35% cited safety concerns, reflecting widespread fear of possible side effects or long-term health risks. In addition, 8% doubted the effectiveness of the vaccine and expressed doubts about its ability to prevent infection or serious illness. Personal beliefs, which include a general scepticism towards vaccines or a mistrust of health authorities, were decisive for 27% of respondents, while 14% cited other reasons such as logistical problems or misinformation. These findings emphasise the need to combat vaccine hesitancy by addressing misinformation and safety concerns, particularly in populations where these issues are most prevalent. Safety concerns are repeatedly cited in research as the main reason for vaccine hesitancy [45]. Misinformation about vaccine ingredients, adverse effects and exaggerated risks exacerbate these concerns, so it is important to conduct targeted information campaigns. Such campaigns should focus on presenting transparent, evidence-based data on the safety and efficacy of vaccines in a culturally sensitive and accessible manner to effectively reach hesitant individuals.

In addition, doubts about the efficacy of the vaccine must be considered. Evidence from similar studies [45] suggests that the publication of real-world data demonstrating the effectiveness of vaccines in preventing serious illness and hospitalisation can mitigate this scepticism. For individuals citing personal beliefs or general distrust, community engagement strategies that promote dialogue and address individual concerns are critical. Engaging trusted local leaders or healthcare providers can help build trust and address misconceptions in a way that resonates with hesitant populations. Finally, to address the "other reasons" such as logistical barriers, practical solutions such as mobile vaccination centres, extended clinic hours and simplified registration procedures are needed. Combining these efforts with education campaigns tailored to the needs of specific communities can increase immunisation coverage and close gaps in care. Together, these strategies represent a comprehensive approach to reducing vaccine hesitancy and ensuring broader public health protection.

To summarise, several Greek studies confirm our findings and suggest that socioeconomic inequalities and health status significantly influence the willingness to be vaccinated against COVID-19. A joint study from Greece and Cyprus found that people from low-income and semi-urban areas were more reluctant to get vaccinated, which is consistent with our findings [46]. This reluctance is often due to limited access to reliable information, mistrust of health authorities or logistical barriers such as transport or scheduling difficulties. Addressing these issues through community outreach programmes and improving access to vaccination centres can mitigate inequalities. Similarly, people with chronic conditions showed a greater willingness to be vaccinated as they perceived a higher risk of severe COVID-19 complications. This highlights the need for targeted education campaigns that emphasise the benefits of vaccination for high-risk groups, not only to protect individuals but also to reduce the overall burden on healthcare systems [47]. Efforts should focus on delivering culturally appropriate and accessible health messages, particularly in areas with low immunisation coverage. Taken together, these findings emphasise the importance of considering both socioeconomic inequalities and chronic diseases when designing public health interventions. Tailored strategies that address the specific needs and barriers of vulnerable populations can improve immunisation coverage, build confidence and ultimately contribute to more equitable health outcomes.

Limitations

Despite the significant contribution of this study to understanding the factors influencing attitudes towards COVID-19 vaccination in Western Attica, some limitations should be noted. First, while there is no direct evidence of self-report bias, it is important to be aware that responses on sensitive topics, such as attitudes towards vaccination, may be subject to social desirability or recall bias. Moreover, the initial sample size of 269 participants was constrained by challenges in recruitment during the pandemic, including psychological stress, limited willingness to participate, and communication barriers. While this allowed the study to proceed, we acknowledge it as a limitation and recommend future research address these challenges with formal sample size calculations and enhanced recruitment strategies. 

Second, there was a loss of participants between the first and second phases of the study, as 14 participants (5.2%) declined to participate in the second phase. Although this attrition rate is relatively low for a multi-phase study, it may have affected the generalisability of longitudinal comparisons. Future studies could use strategies such as regular reminders or incentives for participants to minimise the dropout rate.

Third, the sampling method used in this study was not randomised, but was based on convenience sampling via online platforms. Whilst this approach facilitated access to the target population, it resulted in an overrepresentation of participants with higher levels of education and a lower proportion of people undergoing systematic therapy. This imbalance may limit the generalisability of the findings to broader populations or underrepresented subgroups. Nevertheless, the trends observed provide valuable insights into the key influences on attitudes towards vaccination, particularly among educated and health-conscious individuals.

Finally, although the study used a one-way ANOVA for group comparisons, normality was assessed visually rather than through formal statistical tests. Although this approach is acceptable in exploratory research, it may compromise the robustness of the findings. Future studies should use formal normality tests and consider additional statistical procedures to validate group differences.

Furthermore, the cohort nature of the study, which involved following participants over two phases, prevents causal inferences. Although the analysis allows a detailed examination of correlations and trends within each phase, future longitudinal studies are needed to establish causal relationships between sociodemographic factors and vaccination attitudes. These limitations should be taken into account when interpreting the findings and transferring them to other contexts.

## Conclusions

This study shows the influence of socioeconomic factors and chronic health conditions on COVID-19 vaccination behaviour in Western Attica. Higher income and higher education levels were strongly associated with higher vaccination rates, emphasising the need for targeted interventions to support low-income and less educated populations. Tailored campaigns and incentive-based strategies could be effective in improving immunisation coverage in these groups. In addition, participants with chronic conditions had higher vaccination rates, emphasising the importance of prioritising high-risk groups in public health initiatives. The observed change in attitude among initially reluctant individuals after vaccination emphasises the important role of personal experience in overcoming vaccine hesitancy. This finding emphasises the need for continued community engagement and transparent, evidence-based communication from healthcare providers.

The study provides valuable insights into the complexity of vaccine hesitancy and the role of socioeconomic inequalities in shaping vaccination behaviour. To address the barriers highlighted in this study, we recommend implementing community-based outreach programs, simplifying access to vaccination services, and designing tailored public health campaigns. Additionally, providing financial or social incentives and training healthcare providers in culturally sensitive communication could significantly enhance vaccine uptake in socioeconomically disadvantaged populations. Future research should evaluate the effectiveness of these interventions in diverse contexts.

## Appendices

Appendix 1

**Table 7:** Statistical Analysis of Mean Differences Between Phase 1 and Phase 2 Results are derived from paired t-tests evaluating the mean differences in responses between Phase 1 and Phase 2. P-values < 0.05 indicate statistically significant differences

Socioeconomic Group	T-Statistic	P-Value
Income below 1,500 €	-5,46	0,004
Income over 1,501 €	-9,92	0,022
Primary & Secondary Education	-7,89	0,042
Tertiary Education	-15	0,006
Retirees / Unemployed	-11,52	0,001
Employed	-7,89	0,004

Appendix 2

**Table 8:** Results of the one-way ANOVA comparing citizens' attitudes and perceptions toward vaccines by education level Mean: Average response score. SD: Standard Deviation, F-value: is the ratio of the variance between groups to the variance within groups, P-value: is the Probability Value where a p value < 0.05 was considered statistically significant

Vaccine Attitudes and Perceptions	Educational level				F	P
	Primary School	Secondary School	Higher Education	Master’s degree / PhD.		
	Mean ± SD	Mean ± SD	Mean ± SD	Mean ± SD		
Perceived safety	3.6 ± 0.5	3.6 ± 0.6	3.7 ± 0.8	3.9 ± 0.6	1.198	0.311
Perceived effectiveness and necessity of vaccines	3.9 ± 0.7	3.5 ± 0.6	3.7 ± 0.7	3.8 ± 0.7	4.296	0.006
Acceptability of vaccine selection and scheduling	3.7 ± 0.6	3.5 ± 0.5	3.7 ± 0.7	3.6 ± 0.8	0.868	0.458
Positive values	3.7 ± 0.8	3.8 ± 0.6	4.1 ± 0.7	4.2 ± 0.6	4.757	0.003
Perceived legitimacy of authorities to mandate vaccinations	3.3 ± 0.7	2.9 ± 0.8	3.1 ± 1.0	3.2 ± 0.9	1.259	0.289
Attitude toward Covid-19 vaccine	3.4 ± 0.7	3.2 ± 0.8	3.4 ± 0.9	3.5 ± 0.9	1.520	0.210

Appendix 3

**Table 9:** Socio-demographic and economic characteristics of the citizens who took part in the survey (second phase) N represents the number of values counted and frequency % represents the percentage value

		N	Frequency %
Gender	Male	87	42.03%
	Female	120	57.97%
	Not answered	0	0.00%
Age groups	18-34	15	7.25%
	35-49	58	28.02%
	50-59	52	25.12%
	60+	82	39.61%
Educational level	Primary education	11	5.31%
	Secondary education	62	29.95%
	Higher education	102	49.28%
	Master's degree/PhD	32	15.46%
Employment status	Employee	181	87.44%
	Unemployed	6	2.90%
	Student (higher ed./Univ.)	8	3.86%
	Retired	11	5.31%
	Student	1	0.48%
Employment sector	Public sector	82	45.30%
	Private sector	86	47.51%
	Self-employed	13	7.18%
Nationality	Greek	207	100%
Monthly income	0	10	4.83%
	500-1000	80	38.65%
	1000-1500	88	42.51%
	1500-2000	17	8.21%
	>2000	12	5.80%
Systemic drug therapy	Yes	60	28.99%
	No	147	71.01%

Appendix 4

**Table 10:** Reasons for not receiving the COVID-19 vaccine during Phases 1 and 2 Values outside the brackets represent the number of individuals, while values inside the brackets represent the percentage of the total sample

Reasons for Vaccine Hesitancy	Dec. 2021 to Jan. 2022 - 1st Phase	June 2022 - 2nd Phase
Safety	180 (67%)	17 (35%)
Efficacy	29 (11%)	4 (8%)
Personal Beliefs	33 (12%)	13 (27%)
Other Reasons / Not Answer	27 (10%)	14 (29%)
Total Individuals	269	48

Appendix 5

**Table 11:** Correlation results between public attitudes and perceptions of vaccines and their attitudes toward the COVID-19 vaccine r represents the Pearson correlation coefficient. p indicates the significance level. All correlations are significant at the α = 0.01 level (two-tailed).

Variables	Perceived Safety	Perceived Effectiveness and Necessity of Vaccines	Acceptability of vaccine selection and scheduling	Positive Values	Perceived legitimacy of authorities to mandate vaccinations	Attitude Toward the COVID-19 Vaccine
Perceived Safety	r = 1	r = .675**	r = .636**	r = .636**	r = .609**	r = .671**
		p = .000	p = .000	p = .000	p = .000	p = .000
Perceived Effectiveness and Necessity of Vaccines		r = 1	r = .683**	r = .719**	r = .640**	r = .665**
			p = .000	p = .000	p = .000	p = .000
Acceptability of vaccine selection and scheduling			r = 1	r = .661**	r = .592**	r = .607**
				p = .000	p = .000	p = .000
Positive Values				r = 1	r = .626**	r = .614**
					p = .000	p = .000
Perceived legitimacy of authorities to mandate vaccinations					r = 1	r = .787**
						p = .000
Attitude Toward the COVID-19 Vaccine						r = 1
